# Rosa1, a Transposable Element-Like Insertion, Produces Red Petal Coloration in Rose Through Altering *RcMYB114* Transcription

**DOI:** 10.3389/fpls.2022.857684

**Published:** 2022-04-29

**Authors:** Maofu Li, Hui Zhang, Yuan Yang, Hua Wang, Zhen Xue, Youwei Fan, Pei Sun, Hong Zhang, Xinzhu Zhang, Wanmei Jin

**Affiliations:** ^1^Institute of Forestry and Pomology, Beijing Academy of Agriculture and Forestry Sciences, Beijing, China; ^2^Key Laboratory of Biology and Genetic Improvement of Horticultural Crops (North China), Ministry of Agriculture and Rural Affairs, Beijing, China; ^3^Beijing Engineering Research Center of Functional Floriculture, Beijing, China; ^4^Key Laboratory of Plant Molecular Physiology, Institute of Botany, Chinese Academy of Sciences, Beijing, China; ^5^Institute of Botany, University of Chinese Academy of Sciences, Beijing, China; ^6^Beijing Engineering Research Center for Deciduous Fruit Trees, Beijing, China

**Keywords:** Rosa1 transposable element-like, *RcMYB114*, transcription, petal color, rose, anthocyanin

## Abstract

Rose (*Rosa* sp.) flowers have a rich diversity of colors resulting from the differential accumulation of anthocyanins, flavonols, and carotenoids. However, the genetic and molecular determinants of the red-petal trait in roses remains poorly understood. Here we report that a transposable element-like insertion (Rosa1) into *RcMYB114*, a R2R3-MYB transcription factor’s promoter region causes its transcription, resulting in red petals. In red-petal varieties, *RcMYB114* is expressed specifically in flower organs, but is absent from non-red varieties. Sequencing, yeast two-hybrid, transient transformation, and promoter activity assays of *RcMYB114* independently confirmed the role of Rosa1 in altering *RcMYB114*’s transcription and downstream effects on flower color. Genetic and molecular evidence confirmed that the Rosa1 transposable element-like insertion, which is a previously unknown DNA transposable element, is different from those in other plants and is a reliable molecular marker to screen red-petal roses.

## Introduction

Rose (*Rosa* sp.) is a widely cultivated plant prized for its ornamental, therapeutic, and cosmetic properties (Marmol et al., 2017; Hibrand Saint-Oyant et al., 2018; Raymond et al., 2018). Flower color is of primary importance in ornamental plants and rose exhibits a diverse array of petal colors. Many studies have sought to uncover the molecular and genetic basis of petal coloration in order to accelerate the selective breeding process. In general, differences in color patterns among flowers are determined by the differential regulation of pigment biosynthesis genes during flower development (Martin and Gerats, 1993). For example, red flower color is conferred by the anthocyanin pigment, the biosynthesis of which has been widely studied (Grotewold, 2006; Carbone et al., 2009; Schaart et al., 2013b; Hsu et al., 2015). Anthocyanin biosynthesis involves many structural genes encode essential biosynthetic enzymes including phenylalanine ammonia-lyase (PAL), cinnamate 4-hydroxylase (C4H), 4-coumarate:coenzyme A ligase (4CL), chalcone synthase (CHS), chalcone isomerase (CHI), flavanone 3-hydroxylase (F3H), dihydroflavonol 4-reductase (DFR), anthocyanin synthase (ANS), and UDP flavonoid glucosyltransferase (UFGT) (Winkel-Shirley, 2001). In particularly, the enzymes DFR, ANS, and UFGT are markers of the late anthocyanin biosynthetic pathway (Winkel-Shirley, 2001; Grotewold, 2006; Tanaka et al., 2008; Wang et al., 2020). The genes encoding these late-stage enzymes are regulated by various R2R3-MYB transcription factor genes such as *AtMYB123*(*TT2*) (Baudry et al., 2004), *FaMYB9/FaMYB11* (Schaart et al., 2013a), *FaMYB10* (Wang et al., 2020), *MlPELAN* and *MlNEGAN* (Yuan et al., 2014), *PavMYB10.1* (Jin et al., 2016), *PsMYB12* (Gu et al., 2019), *VvMYBA2r* and *VvMYBA2w* (Jiu et al., 2021), *CgsMYB6*, and *CgsMYB11* (Lin and Rausher, 2021).

The total numbers of R2R3-MYB transcription factors were different in different plant species. For example, a 70 R2R3-MYB transcription factors were identified in sugar beet (Stracke et al., 2014). In grapevine, 108 R2R3-type MYBs were described and classified (Matus et al., 2008). In *Arabidopsis thaliana*, there are 126 MYBs of the R2R3-type described (Stracke et al., 2001). A total of 285 R2R3-MYB transcription factors were identified in banana (Pucker et al., 2020). According to its phylogenetic relationships and short signature motifs the R2R3-MYBs were classified into different subgroups (Kranz et al., 1998; Dubos et al., 2010). The anthocyanin activating R2R3-MYB transcription factors which belong to subgroup 6, had the signature motif “[R/K]PRPRx[F/L].” The first anthocyanin activating R2R3-MYB transcription factors were identified by Paz-Ares et al. (1987). Then many newly R2R3-MYB transcription factors activating anthocyanin were identified in other plants such as snapdragon, lily, petunia, monkey-flower, peony, moth orchid, strawberry, cheery, apple (Goodrich et al., 1992; Quattrocchio et al., 1999; Borevitz et al., 2000; Spelt et al., 2000; Schwinn et al., 2006; Jin et al., 2016; Wang et al., 2020). In snapdragons (*Antirrhinum majus*), the genes *Rosea1*, *Rosea2*, and *Venosa* regulate petal color intensity and anthocyanin pigmentation (Schwinn et al., 2006). In *Asiatic hybrid* lily (*Lilium asiatica hybrid*), the genes *LhMYB12* and *LhMYB6* regulate anthocyanin pigmentation in tepals, filaments, and styles, and *LhMYB6* also regulates light-induced pigmentation in leaves (Yamagishi et al., 2010). In monkey-flower (*Mimulus* spp.), the genes *PELAN* and *NEGAN* regulate anthocyanin pigmentation in the petal lobe and nectar guide, respectively. *NEGAN* is activated by the NEGAN-MlANbHLH1-WD40 complex *via* autocatalytic feedback, which is required to generate anthocyanin spots. The abnormal expression of *PELAN* leads to yellow petals (Yuan et al., 2014). In the orchid *Phalaenopsis equestris*, the differential expression profiles of R2R3-MYB transcription factors regulate the formation of red flowers, which in the orchid *Phalaenopsis Aphrodite*, overexpression of the *PeMYB2* gene causes anthocyanin accumulation in sepals and petals of white-flowered species, suggesting that in this species, *PeMYB2* is responsible for the fully red flower trait (Hsu et al., 2015).

Gene transcription is influenced by several mechanisms including silencing, rearrangement, and insertion of transposable elements (McClintock, 1950; Brown, 1981; Signor and Nuzhdin, 2018; Nakayama and Kataoka, 2019; Gil and Ulitsky, 2020). Barbara McClintock first predicts that transposable elements (Ac/Ds system), which was a mobile piece of DNA, were present in eukaryotic genomes and her studies showed that transposable elements influenced the color of kernels in maize (McClintock, 1950). Transposable elements can replicate and integrate into different positions of the genome, altering the expression of adjacent genes (McClintock, 1950; Butelli et al., 2012; Drongitis et al., 2019; Niu et al., 2019). Transposable elements can be quite volatile and are able to insert themselves into intergenic regions, promoters, exons, introns, and both the 5′ and 3′ untranslated regions (UTRs) of genes. These insertions can lead to both genetic and phenotypic variation (McClintock, 1950; Elbarbary et al., 2016; Hirsch and Springer, 2017; Niu et al., 2019). Often, environmental factors, such as increased temperatures, alter the number and activity of transposable elements, potentially leading to adaptation (Niu et al., 2019). Therefore, transposable elements have potential to quickly create genetic and phenotypic diversity within a population.

During a field survey, we found an interesting red rose (*Rosa chinensis* ‘Semperflorens’ cv. ‘Slater’s Crimson China’) specimen that contained both red and white flowers on the same branch. The flowers had no other obvious differences, having the same number of petals, pistils, stamens, and sepals. We suspected a transposable element may be affecting the expression of one or more R2R3-MYB transcription factor genes. In this research, we sought to understand the genetic basis of this phenomenon.

## Materials and Methods

### Plant Materials and Growth Conditions

Samples of a red rose (*R. chinensis* ‘Semperflorens’ cv. ‘Slater’s Crimson China’) containing both red and white (mutant) flowers on the same plant were identified and collected. Beijing (116°20′ N, 39°56′ E) has a warm temperate semi humid and semi-arid monsoon climate. Summer is hot and rainy, winter is cold and dry, and spring and autumn are short. The annual average temperature is 10∼12°C. All these rose plants were grown outside under nature cultivation conditions at the Institute of Forestry and Pomology, Beijing Academy of Agriculture and Forestry Sciences, Beijing, China. The plants are pruned each December. Samples of the leaves, stems, styles, and petals were used for gene expression analysis. Petals at different developmental stages were used to assess anthocyanin content (Guterman et al., 2002). The leaves, stems, styles, and petals of the other rose varieties were sampled and quick frozen using liquid nitrogen, and stored in the freezer (−70°C).

### Petal Anthocyanin Quantification and Identification

A pH differential method was used to identify and quantify the total anthocyanin content of petals of both red and white (mutant) ‘Slater’s Crimson China’ roses at stage 4 (Cheng and Breen, 1991; Benvenuti et al., 2004). In order to quantify anthocyanin content, rose petals were ground in liquid nitrogen and 10 mg of petal powder was extracted with 0.1% HCl-methanol solution for 4 h, in the dark, at room temperature. All petal extractions were centrifuged for 20 min at 8500 *g* and all supernatants were filtered using a 0.45-μm membrane. A pH differential method was used to estimate the total content of anthocyanins (Cheng and Breen, 1991; Benvenuti et al., 2004). The absorbances at 510 and 700 nm were determined. The anthocyanin content was calculated using anthocyanin content *A* = (a510-a700) pH 1.0–(a510-a700) pH 4.5. All analyses were performed using three biological replicates.

The anthocyanin contents were quantified and calculated as cyanidin-3,5-*O*-diglucoside equivalents in μg per g FW. In order to identify the anthocyanin present, the extracts were assayed using ultra-high-performance liquid chromatography–mass spectrometry (UPLC-MS/MS) using a Acquity UPLC system (Waters, MA, United States) coupled to a XEVO-TQ triple-quadrupole mass spectrometer (Waters, Milford, MA, United States) with electrospray ionization (ESI). The analysis conditions were as follows: a 0.4 mL/min flow rate and positive ion ESI modes, 3.0 kV capillary voltages, and 16 L/h nebulization nitrogen flow. The chromatographs were plotted and analyzed using Origin software (OriginLabs, Northampton, MA, United States). The characteristics of UV-Vis spectra of peaks and the mass spectrometric information of the petal anthocyanin compounds were analyzed according to the difference of the retention times of standards. The anthocyanins content’s relative quantification was analyzed by calculating the peak areas of samples according to the corresponding standard compound’s intensity. All analyses were performed using three biological replicates.

### cDNA and Genomic Sequence Amplification and Sequencing

Genomic DNA was isolated from 250 mg fresh leaf samples from red and white (mutant) with a Super Plant Genomic DNA Kit (Tiangen Biotech Co., Beijing, China). Total RNA extractions of both red and white (mutant) ‘Slater’s Crimson China’ roses at stage 4 were performed using an RNA isolation kit (Tiangen Biotech Co., Beijing, China). After the RNA extract had been treated with DNase I, first-strand cDNA was synthesized with a Revert Aid First-Strand cDNA synthesis kit (Thermo Scientific Inc., Waltham, MA, United States). Primer synthesis was performed by Shanghai Sangon (Sangon, Shanghai, China) (Supplementary Table 3). cDNA sequence *RcMYB114* was cloned using the red petal cDNA as template. The genomic sequence of *RcMYB114* was cloned using the Genomic DNA as template from wild type and mutant petals. PCR was conducted in a 50 μL volume containing 5 μL 10 × buffer, 5 μL dNTPs (2 mmol), 3 μL MgSO4 (25 mmol), 1.5 μL of each primer (10 pmol), 1 U KOD plus polymerase (Toyoboco, Ltd. Life Science Department, Osaka, Japan), and 3 μL genomic DNA (100 ng) or cDNA (100 ng). The cycling conditions were as follows: 1 cycle at 94°C for 4 min, 35 cycles at 98°C for 30 s, 55 ∼58°C for 30 s, and 68°C for 2 min; followed by a final cycle at 68°C for 5 min. PCR products were separated. The amplifying fragments were ligated into pLB-Simple Vector (Tiangen Biotech Co., Beijing, China), transformed into *Escherichia coli* strain, and sequenced.

### Sequence Alignment and Phylogenetic Analysis

The evolutionary analysis of *RcMYB114^Red^* gene was carried out using its protein sequence. The other MYB transcription factor’s protein sequences were acquired from GenBank,^1^ including *A. thaliana* (AtMYB4, AtMYB75, AtMYB90, AtMYB105, AtMYB114) (Kranz et al., 1998; Borevitz et al., 2000; Jin et al., 2000; Stracke et al., 2001), *Solanum pennellii* (SpMYB114) (Kiferle et al., 2015), *A. majus* (AmROSEA1, AmROSEA2, AmVENOSA) (Schwinn et al., 2006), *Malus domestica* (MdMYB16, MdMYB17, MdMYB111, MdMYB114) (Lin-Wang et al., 2010; Xu et al., 2017; Song et al., 2019), *Prunus avium* (PaMYB114) (Jin et al., 2016), *Prunus persica* (PpMYB114) (XP_020420992), *Prunus mume* (PmMYB114) (XP_016652295), *Pyrus* × *bretschneideri* (PbMYB114) (Yao et al., 2017), *Rosa rugosa* (RrMYB114) (QEV87523), *Fragaria vesca* (FvMYB114) (XP_004288854), *Fragaria* × *ananassa* (FaMYB1, FaMYB5, FaMYB9, FaMYB11) (Paolocci et al., 2011; Schaart et al., 2013a), *R. chinensis* (RcMYB23, RcMYB308, RcMYB4, RcMYB113, RcMYB105), *Rosa hybrid* (RhMYB10) (Lin-Wang et al., 2010). The MYB protein alignment was performed using CLUSTALW^2^ (Chenna et al., 2003; Larkin et al., 2007). An evolutionary tree was produced using MEGA X^3^ by the neighbor-joining approach executing 1000 bootstrap replicates (Tamura et al., 2011; Kumar et al., 2018).

### RNA-Seq Analysis of Transcription Levels

Total RNA extractions of both red and white (mutant) ‘Slater’s Crimson China’ roses at stage 4 were performed using an RNA isolation kit (Tiangen Biotech Co., Beijing, China). After the total RNA extraction and DNase I treatment, magnetic beads with Oligo (dT) are used to isolate mRNA. Mixed with the fragmentation buffer, the mRNA is fragmented into short fragments. Than the cDNA was synthesized using the mRNA fragments as templates by reverse transcriptase (Invitrogen, Carlsbad, CA, United States). The sequencing libraries were prepared using the Library Prep Kit (New England BioLabs, Rowley, MA, United States) Short fragments are purified and resolved with EB buffer for end reparation and single nucleotide A (adenine) addition. After that, the short fragments are connected with adapters. After agarose gel electrophoresis, the suitable fragments are selected for the PCR amplification as templates. At last, the library could be sequenced using the HiSeqTM 2000 system (Illumina, San Diego, CA, United States) by Novogene (Novogene Biotech Co., Ltd., Beijing, China). Primary sequencing data that produced by Illumina HiSeqTM 2000, called as raw reads. Raw data (raw reads) of fastq format were firstly processed through in-house perl scripts. In this step, clean data (clean reads) were obtained by removing reads containing adapter, reads containing N base and low quality reads from raw data. At the same time, Q20, Q30, and GC content the clean data were calculated. All the downstream analyses were based on the clean data with high quality. After QC, clean reads was aligned to the reference sequences with SOAPaligner/SOAP2. The alignment data is utilized to calculate distribution of reads on reference genes and perform coverage analysis. The gene expression level is calculated by using RPKM method (Mortazavi et al., 2008). The RPKM method is able to eliminate the influence of different gene length and sequencing discrepancy on the calculation of gene expression. Therefore, the calculated gene expression can be directly used for comparing the difference of gene expression among samples. Differential expression analysis was performed using the edgeR (Robinson et al., 2010). The *P* values were adjusted using the Benjamini and Hochberg method. Corrected *P*-value 0.05 and absolute fold change of 2 were set as the threshold for significantly different expression.

### Real-Time Quantitative PCR Assay of Genes Related to Anthocyanin Biosynthesis

Total RNA of each tissue sample was extracted and first-strand cDNA was synthesized with a cDNA synthesis kit (Thermo Scientific, Waltham, MA, United States). RT-qPCR was performed using a Bio-Rad CFX96 system (Bio-Rad, California, CA, United States). For RT-qPCR conditions, 10 μL reaction mixture included 5 μL 2 × SYBR Premix, 1 μL forward primer (10 μM), 1 μL reverse primer (10 μM), 1 μL cDNA template (20 ng) and 2 μL ddH_2_O; the PCR conditions were as follows: 1 cycle at 95°C for 30 s, 40 cycles at 95°C for 5 s, and 1 cycle at 60°C for 30 s. Primers for various regulatory and structural genes related to the anthocyanin biosynthesis pathway are shown in Supplementary Table 3. Data were analyzed using the 2^–ΔΔ*CT*^ method as outlined by Livak and Schmittgen (2001). Expression of specific genes were normalized to *actin* (KC514920) (Meng et al., 2013). All analyses were performed using three biological replicates.

### Yeast Two-Hybrid Assay of Gene Function

Yeast Two-Hybrid (Y2H) experiments were performed according to the method used in a previous study. Briefly, AH109-competent cells were co-transformed according to the manufacturer’s instructions (Clontech Laboratories, California, CA, United States). *RcMYB114^Red^* and *RcWD40* were introduced into *pGADT7* to produce fusion proteins using the GAL4 activation domain (AD). *RcMYB114^Red^* and *RcWD40* were separately cloned into *pGBKT7* to make recombinants with the GAL4 DNA binding domain (BD). The vector of *RcbHLH* fused with the GAL4 AD and BD was kept in our laboratory (Li et al., 2017). All primers are listed in Supplementary Table 3. All constructs were confirmed by enzyme digestion and sequencing. The various combinations of BD and AD vectors were co-transformed into yeast strain AH109 using the lithium acetate method (Gietz et al., 1995) and selected on SD/–Leu–Trp media under 30°C culture conditions for 3–4 days. To assay the interaction, these clones were then incubated on SD/–Ade–His–Leu–Trp culture media under 30°C culture conditions for 7 days. β-galactosidase tests were performed on the same plate and positive clones were dyeing by using 3–5 μL 4 mg/mL X-α-gal, and false-positive activation was excluded using the P53 plus SV40 vector.

### *Nicotiana benthamiana* Expression Assay of Gene Function

Transient expression of *RcMYB114^Red^* constructs was performed using a Hyper *Trans* system (Sainsbury et al., 2009; Butelli et al., 2017). Briefly, *RcMYB114^Red^* was isolated from the genomic DNA of both the white (mutant) and red flowers of ‘Slater’s Crimson China.’ *RcMYB114^Red^* was placed into the pEAQ686HT-DEST1 vector. The *RcMYB114^Red^* plasmid was transformed into *Agrobacterium tumefaciens* GV3101. The transformation protocol was conducted as previously described (Sparkes et al., 2006). Leaves were sampled 7 days after injection. We then photographed the leaves and carried out measurement of the total anthocyanin content, high-performance liquid chromatography analysis of anthocyanin compounds, and RT-qPCR analysis of the expression of the *RcMYB114^Red^* transcription factor and other genes.

### Chromatin Immunoprecipitation Assay

Chromatin immunoprecipitation assays were performed according to Bowler’s methods (Bowler et al., 2004). Briefly, we used a rabbit (New Zealand) to produce an IgG antibody to RcMYB114*^Red^*. The rabbit IgG was purified using Pan’s protocol (Pan et al., 2005). The chromatin immunoprecipitation experiments were carried out as described by Bowler (Bowler et al., 2004) using a Pierce Agarose ChIP kit (No. 26156, Thermo Scientific, Waltham, MA, United States). Primers were designed according to the promoter sequences of *RcPAL*, *RcC4H*, *RcCHS*, *RcCHI*, *RcF3H*, *RcFLS*, *RcLAR*, *RcDFR*, *RcANS*, and *RcUFGT.*

### *pRcMYB114^Red^* and *pRcMYB114^White^* Promoter-*β-Glucuronidase* Fusion Gene Transformation and Histochemical β-Glucuronidase Assay

We fused the *pRcMYB114^Red^* and *pRcMYB114^White^* promoters to the β-Glucuronidase (*GUS*) gene vector, which were subsequently injected into *Nicotiana benthamiana* leaves (Sainsbury et al., 2009; Butelli et al., 2017). Leaves were sampled 7 days after injection and soaked in X-Gluc buffer (12 mM potassium ferricyanide, 12 mM potassium ferrocyanide, 0.3% (v/v) Triton X-100, and 1 mg/ml 5-bromo-4-chloro-3-indolyl-β-D-glucuronide). The buffer was infiltrated into the samples under a vacuum. The leaves were stained overnight at 37°C, washed in 70% (v/v) ethanol several times, and then photographed (Koo et al., 2007).

## Results

### *RcMYB114* Shares High Homology With Anthocyanin-Regulating Genes in Many Plants

**FIGURE 1:**
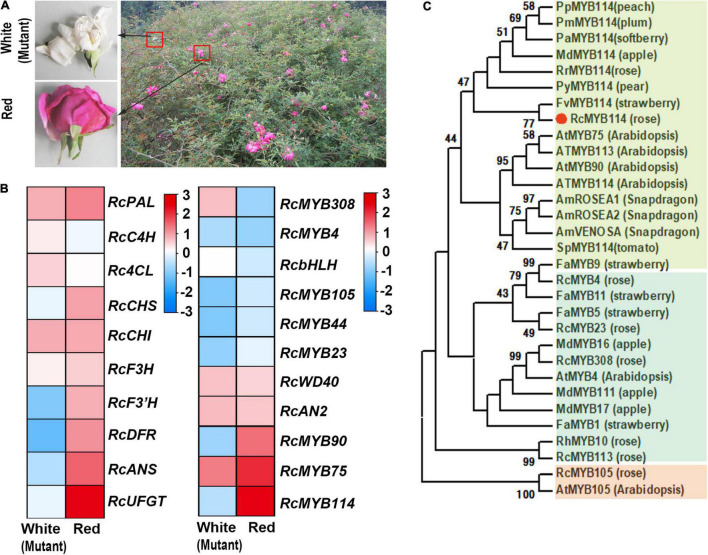
Identification of the gene (*RcMYB114*) regulating anthocyanin biosynthesis in our mutant rose. **(A)** A red rose (*R. chinensis* ‘Semperflorens’ cv. ‘Slater’s Crimson China’) specimen was found to contain both red and white (mutant) flowers. **(B)** RNA-seq heat map of genes related to anthocyanin biosynthesis. **(C)** Evolutionary analysis of MYB genes.

During a field survey, we found a red rose (*R. chinensis* ‘Semperflorens’ cv. ‘Slater’s Crimson China’) specimen that contained both red and white (mutant) flowers on the same branch (Figure 1A). We collected both red and white flowers from this specimen and performed an RNA-seq analysis. We found that several key genes upstream of the anthocyanin biosynthesis pathway, including *RcPAL*, *RcCHI*, *RcCHS*, and *RcC4H*, had significantly higher expression levels in red flowers compared to white flowers. Additionally, several key downstream genes, including *RcDFR*, *RcANS*, and *RcUFGT*, also had significantly higher expression levels in red flowers compared to white flowers (Figure 1B). We further confirmed these results by real-time quantitative PCR (RT-qPCR). Overall, we found 125 *MYB* genes expressed in red flowers and 118 *MYB* genes expressed in white flowers. Among these *MYB* genes, *RcMYB113*, *RcMYB308*, *RcMYB75*, *RcMYB90*, *RcMYB114*, *RcMYB4*, *RcMYB105*, and *RcMYB23* encode R2R3-type MYB transcription factors. The RNA-seq heatmap indicated that the relative transcription levels of these genes showed two distinct expression patterns between red and white flowers. The expression levels of *RcMYB113*, *RcMYB308*, and *RcMYB4* were lower in red flowers compared to white flowers. Conversely, the expression levels of *RcMYB75*, *RcMYB114*, *RcMYB105*, and *RcMYB23* were higher in red flowers compared to white flowers. Notably, *RcMYB114* was highly expressed in red flowers, but was absent in white flowers (Figure 1B). Based on gene annotation analysis, *RcMYB114* was mapped to chromosome 7 (GenBank accession: MW239569). BLAST similarity analysis indicated that *RcMYB114* was identical to the RchiOBHmChr7g0235271.^4^ Further, SMART analysis showed that *RcMYB114* encoded an R2R3-MYB transcription factor.^5^

To determine the phylogenetic relationship of RcMYB114 to other known R2R3-MYB transcription factors, we constructed a tree of about 30 closely related transcription factors by the neighbor-joining approach (Figure 1C). We found that the protein product of *RcMYB114* (RcMYB114) clustered with strawberry (*Fragaria* spp.) FvMYB114. The related proteins are known to primarily regulate anthocyanin biosynthesis, including FvMYB114 (*F. vesca*), and it was suspected that RCMYB114 does the same (Figure 1C).

### *RcMYB114* Regulates Anthocyanin Biosynthesis in Roses

**FIGURE 2:**
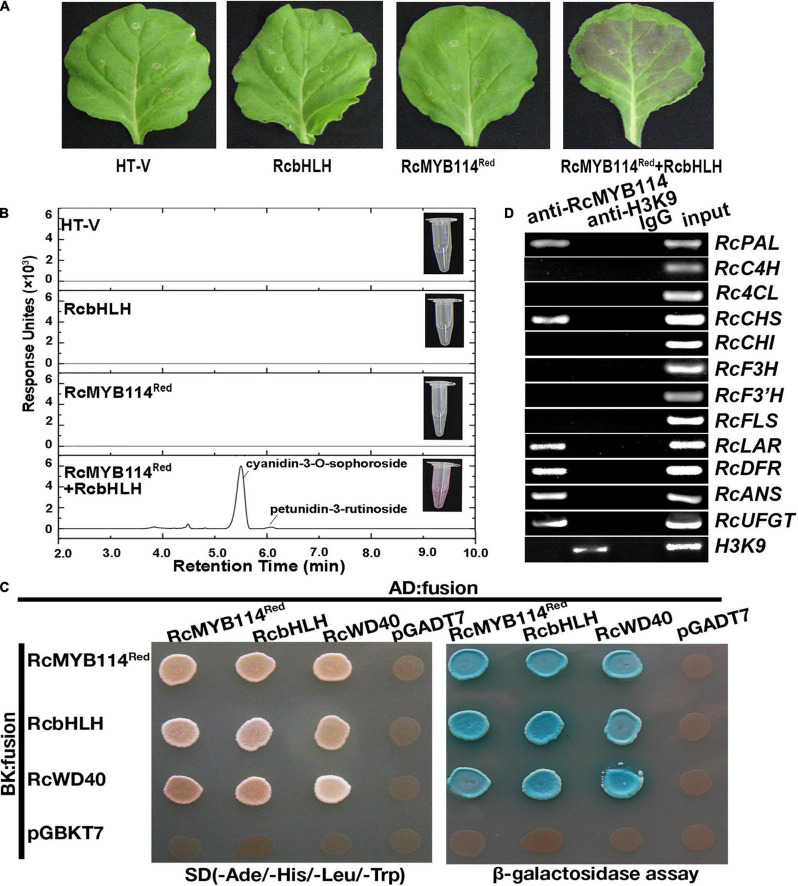
Confirmation of the role of *RcMYB114* in regulating anthocyanin biosynthesis. **(A)** Leaf phenotypes of *N. benthamiana* after being infiltrated with different constructs or the empty vector (HT-V). **(B)** Anthocyanins from the infiltrated *N. benthamiana* leaves were assayed by UPLC-MS/MS. **(C)** The yeast two-hybrid (Y2H) assay to probe the interaction of RcMYB114 with RcWD40 and RcbHLH within the MBW complex. The co-transformants were screened on media (−Ade/−His/−Leu/−Trp). β-galactosidase tests were performed on the same plate and positive clones were dyeing by using 3–5 μL 4 mg/mL X-α-gal, and false-positive activation was excluded using the P53 plus SV40 vector. **(D)** RcMYB114 was selectively recruited to *RcPAL*, *RcCHS*, *RcLAR*, *RcDFR*, *RcANS*, and *RcUFGT* promoter regions as determined by the ChIP assay. Anti-H3K9 was used as positive control and Anti-IgG was negative control.

We cloned *RcMYB114* using the genomic DNA as template from both white mutant petals and red wild-type petals. The results found that the sequences of *RcMYB114^red^* and *RcMYB114^white^* were identical. To confirm whether *RcMYB114* is responsible for regulating anthocyanin biosynthesis, a Hyper *Trans* expression vector was used to transiently deliver the *RcMYB114^Red^* plus *RcbHLH* into *N. benthamiana* leaves. We found that, after 5 days, leaves successfully infiltrated with *RcMYB114^Red^* plus *RcbHLH* showed red color (Figures 2A,B). We found that the pigmentation was mainly comprised of cyanidin-3-*O*-sophoroside, and the total anthocyanin content of these leaves was 57.92 mg/100 g fresh weight (FW).

To further verify the role of *RcMYB114*, yeast two-hybrid (Y2H) and chromatin immunoprecipitation (ChIP) assays were carried out. The classical MBW complex, which acts as a regulatory hub for the anthocyanin biosynthesis and other processes, consists of MYB, basic helix-loop-helix (bHLH), and WD40 proteins. In the Y2H assay, yeast system vectors were constructed using RcMYB114*^Red^*, RcWD40, and RcbHLH. We observed that all three MBW complex proteins interacted with each other in yeast cells (Figure 2C). ChIP analysis illustrated that *RcMYB114* selectively bound the *RcPAL*, *RcCHS*, *RcLAR*, *RcDFR*, *RcANS*, and *RcUFGT* promoter regions containing the MYB binding site (AACCTAA) for light-responsive elements (Figure 2D). These results indicate that *RcMYB114* encodes a transcription factor protein that interacts with RcWD40 and RcbHLH and is selectively recruited to *RcPAL*, *RcCHS*, *RcLAR*, *RcDFR*, *RcANS*, and *RcUFGT* promoter regions to regulate anthocyanin biosynthesis and accumulation.

### A Fragment Insertion (Rosa1) in the Promoter Region of *Rcmyb114* Causes Its Expression in Red Rose

**FIGURE 3:**
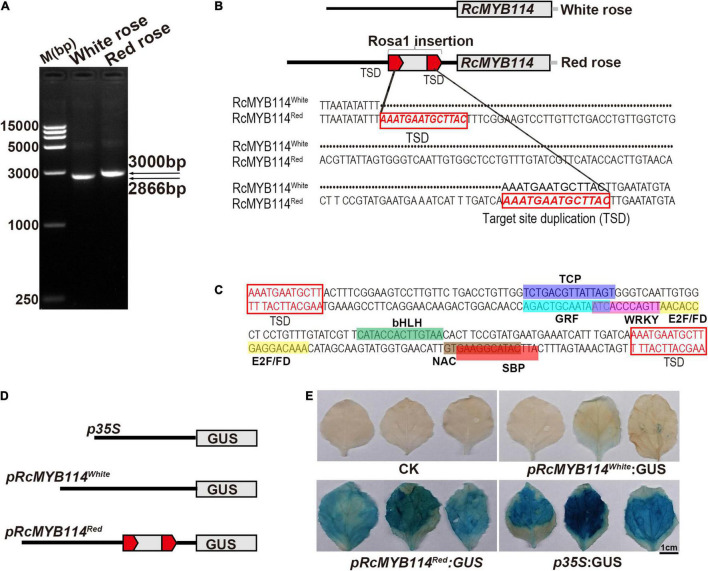
Confirmation that the Rosa1 transposable element-like sequence inserted into the promoter region of *RcMYB114* alters its expression. **(A)** Promoters, *pRcMYB114^Red^* and *pRcMYB114^White^*, were cloned by PCR amplification. **(B)** Sequence analysis of Rosa1, a transposable element-like sequence in the promoter of *RcMYB114^Red^*. Red frame and letters indicate the target site duplication (TSD) and sequence. **(C)** Domain analysis of the Rosa1 transposable element-like sequence. **(D)** Schematic diagram of the *pRcMYB114^Red^* and *pRcMYB114^White^* promoter-β-Glucuronidase (*GUS*) fusion vectors. The *RcMYB114* promoters replaced the CaMV35S promoter in pBI121. **(E)** Histochemical assay of GUS activity in *N. benthamiana* leaves after being infiltrated with the *p35S:GUS*, *pRcMYB114*^Red^*:GUS*, and *pRcMYB114*^White^*:GUS* constructs.

We found that *RcMYB114^Red^* and *RcMYB114^White^* were identical in both form and function. We also found that red roses expressed *RcMYB114^Red^* in high quantities while white roses did not express *RcMYB114^White^*. To determine the cause of this dramatic difference in transcript abundance between red and white roses on the same plant, we isolated the upstream promoters of *RcMYB114^Red^* and *RcMYB114^White^*. The promoter sequence of *RcMYB114^Red^* (*pRcMYB114^Red^*) was approximately 3 kb, but that of *RcMYB114^White^* was only 2866 bp (Figures 3A,B). After cloning and sequencing these fragments, we found a 148-bp fragment inserted at −758 bp upstream of the ATG start codon of *RcMYB114^Red^*, which was absent in *RcMYB114^White^*. The 148-bp fragment was named Rosa1 and contained a *cis*-acting element binding site for transcription factors. For example, the sense chain had bZIP, TCP, and bHLH domains and the antisense chain had GRF, WRKY, E2F/PD, NAC, and SBP domains (Figure 3C). The element CATTCATACGGAAGTG of SBP is the binding site for MYB transcription factors, which are involved in regulation of flavonoid biosynthesis (Solano et al., 1995). There are seven chromosomes in haploid roses, and we found that the Rosa1 fragment is found in 5–8 locations, distributed across all chromosomes. Rosa1 is mainly inserted in the promoter, 5′UTR, 3′UTR, intron, and intergenic regions of transcription factor genes, including MYB, TIFY, and WD40, and other genes related to growth and development, including zinc finger, wuschel family, adenyltransferase, and CoA reductase (Supplementary Table 1).

To investigate whether the Rosa1 inserted in the promoter region of *RcMYB114^Red^* alters its expression, we used the GUS reporter system. We constructed *pRcMYB114^Red^* (3 kb, including Rosa1) and *pRcMYB114^White^* (2866 bp, without Rosa1) promoter-*GUS* fusion constructs (Figure 3D). The *RcMYB114* promoter-*GUS* fusion vectors were infiltrated into the abaxial surface of *N. benthamiana* leaves. After GUS staining, the *35S:GUS* construct showed the strongest expression, followed by the *pRcMYB114*^Red^* GUS* construct, with the *pRcMYB114*^White^* GUS* construct having the weakest expression. This experiment confirmed that Rosa1 does alter gene expression (Figure 3E).

**FIGURE 4:**
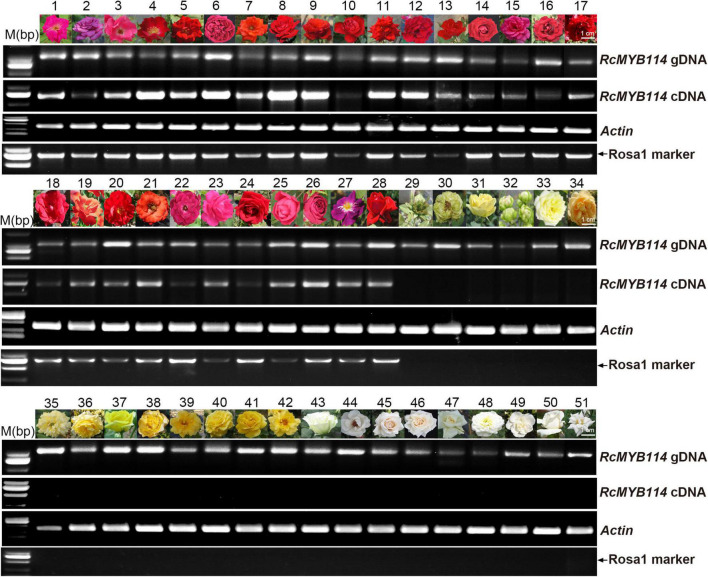
Expression of *RcMYB114* across different petal-color phenotypes is dependent on the presence of Rosa1 transposable element-like sequence within the upstream regulatory sequence of *RcMYB114.* PCR amplification of *RcMYB114* from genomic DNA and petal cDNA of 51 rose varieties. *Actin* was used as a loading control. Only the red-petal varieties contained the Rosa1 transposable element-like sequence.

Next, we analyzed the *RcMYB114* gene and its upstream sequence in different rose varieties (red, yellow, white, and green) (Supplementary Table 2), to confirm the universal existence of this transposable element-like sequence and how it is related to *RcMYB114* transcript levels and petal color across phenotypes. *RcMYB114* was amplified from the genomic DNA of 51 rose varieties (Figure 4). All the tested red-petal varieties showed high expression of *RcMYB114*, including ‘Slater’s Crimson China,’ ‘Blue River,’ ‘Betty Prior,’ ‘Dortmund,’ ‘Uncle Walter,’ ‘Pierre de Ronasard,’ ‘Hiohgi,’ ‘Hohoemi,’ ‘Red Success,’ ‘Terrazza Voila,’ ‘Wonderful Wife,’ ‘Crimson Glory,’ ‘Red Cap,’ ‘Carola,’ ‘Seba,’ ‘Black Lady,’ ‘Gold Carriage,’ ‘Zajibiaoyan,’ ‘Hana-Busa,’ ‘Huangjiabaxinuo,’ ‘2018-08-3,’ ‘Burgundy Iceberg,’ ‘Xiangchun,’ ‘Dongfanghong,’ ‘Cherry Bonica,’ ‘Red Lace,’ ‘Rhapsody in Blue,’ and ‘Ingrid Bergman.’ *RcMYB114* was not expressed in the non-red varieties (green, yellow, and white), including the green varieties ‘Viridiflora,’ ‘Green Star,’ ‘Lvye,’ ‘Éclair,’ and ‘Creamy Eden’; the yellow varieties ‘Golden Celebration,’ ‘Yellow Meilove,’ ‘Adolf Horstmann,’ ‘Golden Scepter,’ ‘Kent Princess,’ ‘Oregold,’ ‘Gold Bunny,’ ‘Australian Gold,’ and ‘Golden Shower’; and the white varieties ‘Baihe,’ ‘2018-31-117,’ ‘White Ohara,’ ‘Bridal White,’ ‘Lvyun,’ ‘Beizhi,’ ‘Snowflake,’ ‘White Satin,’ and ‘Tiantanbai’ (Figure 4). All the red-petal varieties contained the Rosa1 sequence in the promoter of their *RcMYB114* gene, and all non-red varieties lacked Rosa1 (Figure 4). This suggests that the Rosa1 insertion in the upstream regulatory sequence of *RcMYB114* altered *RcMYB114* expression, resulting in anthocyanin biosynthesis accumulation, and was therefore responsible for the red-petal phenotype in rose.

### A Regulatory Model for Anthocyanin Biosynthesis and Accumulation in Rose Petals

**FIGURE 5:**
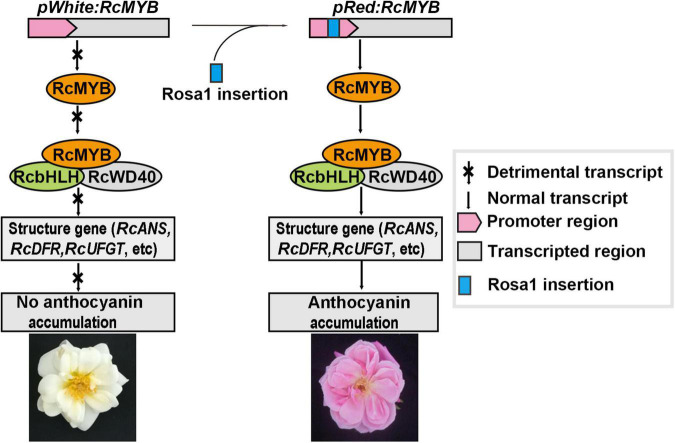
A regulatory model for anthocyanin biosynthesis and accumulation in rose petals.

Rose plants show a rich diversity of flower colors. The formation of petal color depends on the differential activation of pigment biosynthesis genes and accumulation of pigments during flower development. Red petal color is conferred by anthocyanins, and the *RcMYB114* transcription factor regulates anthocyanin biosynthesis and accumulation in rose. A Rosa1 fragment insertion can change gene expression by altering transcription. In white, green, and yellow rose varieties, *RcMYB114* is not expressed without the Rosa1 fragment insertion during flower development. Without *RcMYB114* transcription and translation, a putative canonical MBW complex can’t form, which is responsible for the expression of the key anthocyanin structural genes, such as *RcDFR*, *RcANS*, and *RcUFGT*. Therefore, anthocyanin biosynthesis and accumulation are blocked, resulting in white-, green-, and yellow-petal roses. However, the Rosa1 fragment insertion in the upstream regulatory sequence of *RcMYB114* causes its expression during flower development. RcMYB114 is then available to form the canonical MBW complex with RcWD40 and RcbHLH. This promotes the expression of *RcDFR*, *RcANS*, *RcUFGT*, and other downstream anthocyanin structural genes, eventually leading to the accumulation of anthocyanins and red petal color in rose flowers (Figure 5).

## Discussion

### R2R3-MYB Family Genes Are Critical Regulators of Anthocyanin Accumulation in Plants

Anthocyanins are secondary metabolites that have multiple biological functions in plants (Falcone Ferreyra et al., 2012). The R2R3-MYB transcription factors, regulate the anthocyanin biosynthesis pathway, thus effecting anthocyanin biosynthesis and accumulation (Hichri et al., 2011; Jin et al., 2016; Andersen et al., 2019; Gu et al., 2019; Li et al., 2019; Wang et al., 2020; Jiu et al., 2021; Lin and Rausher, 2021; Yin et al., 2021). The addition or deletion of sequences in the coding region of R2R3-MYB genes can affect their transcription and protein synthesis and ultimately alter the accumulation of anthocyanins. For example, in strawberry (*Fragaria* × *ananassa* Duch.), *FaMYB10* plays an important role in controlling anthocyanin biosynthesis. An 8-bp ACTTATAC insertion at the C terminus genomic region, leads to a code shift mutation and produces the white octoploid strawberry (Wang et al., 2020). In sweet cherry (*P. avium* L.), the inheritance of cherry fruit skin color is regulated by a single gene involved in anthocyanin biosynthesis, *PavMYB10.1*. This gene has three different alleles: *PavMYB10.1a*, *PavMYB10.1b*, and *PavMYB10.1c*. *PavMYB10.1a* contains an intact 672-bp cDNA sequence, conferring red skin color. A 1-bp deletion in *PavMYB10.1b* confers blush skin color and an insertion/deletion (indel) in the same sequence region of *PavMYB10.1c* confers yellow skin color (Jin et al., 2016).

Although different genes control fruit coloration and flower coloration, their regulation mechanisms are very similar. In petunia (*Petunia* × *atkinsiana*), the *anthocyanin2* (*an2*) locus, which encodes an R2R3-MYB regulator, is a significant regulator of petal limb color. Other petunia species, including *P. integrifolia* and *P. axillaris*, possess several color variants. The *an2* allele has two alternative code shifts through insertion into one position, which cause an *an2* functorial defect and alter the flower color (Quattrocchio et al., 1999). In snapdragons (*Antirrhinum* spp.), the *Rosea* locus, which includes *Rosea1*, *Rosea2*, and *Venosa* MYB-related transcription factors, has three different alleles that regulate the intensities and patterns of magenta anthocyanin pigmentation in petal. Wild-type petals are nearly wholly colored and contain a high concentration of magenta anthocyanin in the corolla. Two mutant alleles (*ros^col^* and *ros^dor^*) at the *Rosea* locus are created by indels, resulting in a low level of anthocyanin presentation confined to the petals’ inner epidermis or a low level anthocyanin presentation toward tube’s base and anthocyanin presentation on dorsal lobes’ outer epidermis, respectively (Schwinn et al., 2006). We found that the *RcMYB114* gene can affect the accumulation of anthocyanins in roses. According to the analysis results of different varieties with different petal colors, at the genomic level, *RcMYB114* gene was amplified in the genomic DNA of all rose varieties. Meanwhile, at the level of gene transcription, the *RcMYB114* was only expressed in all red petal variety, but not in non-red rose varieties such as yellow, green and white petals. Also, we found that the transcription level of *RcMYB114* was different in all red varieties, and some varieties had very low expression. These results indicated that there are other MYB genes that cooperate with *RcMYB114* and form MYBs regulation networks to determine the red color of rose petals.

### Rosa1 Plays a Critical Role in Determining Flower Color by Altering the Transcription of R2R3-MYB Genes

Insertion incidents influence nearby gene transcription and raise the mutation rate near the insertion site, leading to the diversification of plant traits (Bennetzen, 2000; Elbarbary et al., 2016; Chuong et al., 2017; Niu et al., 2019; Jo and Kim, 2020). For example, in apple (*M. domestica* Borkh.), *MdMYB1*, which is related to the anthocyanin biosynthesis pathway, regulates fruit red skin phenotype. The *MdMYB1* gene possesses three different alleles: *MdMYB1-1*, *MdMYB1-2*, and *MdMYB1-3*. The *MdMYB1-1* allele is dominant and leads to anthocyanin biosynthesis and red fruit skin. The other two alleles, *MdMYB1-2* and *MdMYB1-3*, lead to limited anthocyanin biosynthesis, producing non-red fruit skin. There is a 4097-bp retrotransposon insertion with two target site duplications upstream of the *MdMYB1* promoter region, which controls the development of red skin color in apple (Zhang et al., 2019). Whether this insertion is present or not is stably transferred from one generation to the next (Zhang et al., 2019). In citrus, *Ruby*, an R2R3-MYB gene, regulates fruit color. A Copia-like retrotransposon inserted into *Ruby* induces its expression, resulting in the striking red color of Sicilian blood oranges (*Citrus sinensis*). In addition, the differential expression of *Ruby* due to various site mutations, deletions, and insertions of transposable elements gives rise to variations in leaf and petal colors in different *Citrus* species and domesticated cultivars (Butelli et al., 2012, 2017). In grape (*Vitis vinifera* L.), red fruit color is closely associated with the expression of *VvMYBA1*, which regulates anthocyanin biosynthesis. Black-skinned cultivars contain a retrotransposon, Gret1, inserted into the upstream region of *VvMYBA1*. Because no *VvMYBA1* transcripts are detected in white-skinned grapes, it can be concluded that the insertion drives expression of *VvMYBA1*, presenting as dark-skinned grapes (Kobayashi et al., 2004; Walker et al., 2007).

The Rosa1 transposable element we found is a previously unknown DNA transposon, and different from those in apple, citrus, and grape. For example, Rosa1 contains *cis*-acting element binding sites for transcription factors: the sense chain contains bZIP, TCP, and bHLH domains, and the antisense chain contains GRF, WRKY, E2F/PD, NAC, and SBP domains. In rose, the Rosa1 transposable element, which inserts into *RcMYB114*’s promoter region, may be considered as an enhancer, promoting the development of red flower color. Cultivars without the Rosa1 transposable element do not effectively produce anthocyanin pigments. Thus, the Rosa1 transposable element can serve as a DNA molecular marker to distinguish red petal roses.

## Conclusion

RcMYB114, a R2R3-MYB transcription factor, shares high homology with anthocyanin-regulating genes in many plants. RcMYB114 is part of an MBW complex and selectively recruited to structure gene’s promoter regions to regulate anthocyanin biosynthesis and accumulation in rose. Rosa1, a148-bp transposable element-like, insertion in the promoter region of *RcMYB114*, enhanced *RcMYB114* transcript level and resulted in upregulation of anthocyanin biosynthesis genes, accumulated anthocyanins. Thus, the Rosa1 can alter gene transcription and produce rose red petal.

## Data Availability Statement

The datasets presented in this study can be found in online repositories. The names of the repository/repositories and accession number(s) can be found below: The raw RNA-seq data were deposited in the CNGB Nucleotide Sequence Archive (https://db.cngb.org/cnsa/) of China National GenBank (CNGB) database (accession number: CNP0001468). The RcMYB114, RcWD40 cDNA, RcbHLH, and Rosa1 are available in the GenBank database under the accession numbers MW239568, MW239571, KY783912, and MW430097.

## Author Contributions

WJ designed research. ML, YY, HW, HoZ, XZ, and ZX performed research. ML, HuZ, YF, and PS analyzed data. WJ and ML wrote the manuscript. All authors contributed to the article and approved the submitted version.

## Conflict of Interest

The authors declare that the research was conducted in the absence of any commercial or financial relationships that could be construed as a potential conflict of interest.

## Publisher’s Note

All claims expressed in this article are solely those of the authors and do not necessarily represent those of their affiliated organizations, or those of the publisher, the editors and the reviewers. Any product that may be evaluated in this article, or claim that may be made by its manufacturer, is not guaranteed or endorsed by the publisher.

## Footnotes

1
https://www.ncbi.nlm.nih.gov/genbank/

2
https://www.genome.jp/tools-bin/clustalw

3
https://www.megasoftware.net/

4
https://lipm-browsers.toulouse.inra.fr/pub/RchiOBHm-V2/

5
http://smart.embl-heidelberg.de/
