# Supplementary material for: Rosa1, a Transposable Element-Like Insertion, Produces Red Petal Coloration in Rose Through Altering RcMYB114 Transcription
Source: Front Plant Sci. 2022 Apr 29;13:857684. doi: 10.3389/fpls.2022.857684 (PMC9100400; doi:10.3389/fpls.2022.857684)
Supplement: Supplementary file 1 [file Data_Sheet_1.docx]

Supplementary Material

# Supplementary Tables

**Supplementary Table S1** Distribution of the Rosa1 transposable element-like on chromosomes and location of genes in rose.

**Supplementary Table S2** Different rose varieties were used in this study.

**Supplementary Table S3** The sequences list of primers used in this study.

**Supplementary Table S****1** Distribution of the Rosa1 transposable element-like on chromosomes and location of genes in rose

| NO | Gene ID | Location in Chromosomes | start | end | Location in genes | Gene describes | Gene sorted | Reference |
| --- | --- | --- | --- | --- | --- | --- | --- | --- |
| 1 | RchiOBHmChr7g0235271 | Chr7 | 1 | 148 | promoter | Transcription factor RcMYB114 | Transcription factor | This study |
| 2 | RchiOBHmChr7g0235371 | [Chr7](https://lipm-browsers.toulouse.inra.fr/pub/RchiOBHm-V2/jbrowse/current/?data=../data/RchiOBHm-V2&loc=RchiOBHmChr7:60095350..60105229&highlight=RchiOBHmChr7:60100350..60100229) | 14 | 135 | promoter | Ribonuclease H-like domain, hAT-like transposase, RNase-H | Transposase | (Bundock and Hooykaas, 2005) |
| 3 | RchiOBHmChr7g0235391 | [Chr7](https://lipm-browsers.toulouse.inra.fr/pub/RchiOBHm-V2/jbrowse/current/?data=../data/RchiOBHm-V2&loc=RchiOBHmChr7:60109363..60119242&highlight=RchiOBHmChr7:60114363..60114242) | 14 | 135 | promoter | Transcription factor/ chromatin remodeling BED-type (Zn) family | Transcription factor | (Job et al., 2016) |
| 4 | RchiOBHmChr6g0281411 | [Chr6](https://lipm-browsers.toulouse.inra.fr/pub/RchiOBHm-V2/jbrowse/current/?data=../data/RchiOBHm-V2&loc=RchiOBHmChr6:44665054..44674933&highlight=RchiOBHmChr6:44670054..44669933) | 14 | 135 | promoter | Hydroxymethylglutaryl-CoA reductase (NADPH) | Reductase | (Doblas et al., 2013) |
| 5 | RchiOBHmChr4g0417531 | [Chr4](https://lipm-browsers.toulouse.inra.fr/pub/RchiOBHm-V2/jbrowse/current/?data=../data/RchiOBHm-V2&loc=RchiOBHmChr4:42828406..42838527&highlight=RchiOBHmChr4:42833406..42833527) | 14 | 135 | promoter | P-loop containing nucleoside triphosphate hydrolase, leucine-rich repeat domain, L | Disease resistance protein | (Ashfield et al., 2004) |
| 6 | RchiOBHmChr2g0135911 | [Chr2](https://lipm-browsers.toulouse.inra.fr/pub/RchiOBHm-V2/jbrowse/current/?data=../data/RchiOBHm-V2&loc=RchiOBHmChr2:53138055..53147934&highlight=RchiOBHmChr2:53143055..53142934) | 14 | 135 | 3’UTR | Anther development,stomium development WUSCHEL family | Transcription factor | (Plong et al., 2021) |
| 7 | RchiOBHmChr2g0121211 | [Chr2](https://lipm-browsers.toulouse.inra.fr/pub/RchiOBHm-V2/jbrowse/current/?data=../data/RchiOBHm-V2&loc=RchiOBHmChr2:34146526..34156400&highlight=RchiOBHmChr2:34151526..34151400) | 14 | 135 | promoter | Laccase | Metal ion binding protein | (Abdel-Ghany and Pilon, 2008) |
| 8 | RchiOBHmChr3g0462251 | [Chr3](https://lipm-browsers.toulouse.inra.fr/pub/RchiOBHm-V2/jbrowse/current/?data=../data/RchiOBHm-V2&loc=RchiOBHmChr3:9969496..9979612&highlight=RchiOBHmChr3:9974496..9974612) | 14 | 131 | 3’UTR | Hypothetical protein | Uncharacterized protein |  |
| 9 | RchiOBHmChr6g0291101 | [Chr6](https://lipm-browsers.toulouse.inra.fr/pub/RchiOBHm-V2/jbrowse/current/?data=../data/RchiOBHm-V2&loc=RchiOBHmChr6:54298842..54308962&highlight=RchiOBHmChr6:54303842..54303962) | 15 | 135 | 3’UTR | Small GTPase superfamily, EF-hand domain pair | GTPase | (Wang et al., 2008) |
| 10 | RchiOBHmChr3g0474261 | [Chr3](https://lipm-browsers.toulouse.inra.fr/pub/RchiOBHm-V2/jbrowse/current/?data=../data/RchiOBHm-V2&loc=RchiOBHmChr3:19917657..19927537&highlight=RchiOBHmChr3:19922657..19922537) | 15 | 135 | 3’UTR | Rho GTPase activation protein | GTPase | (Wang et al., 2008) |
| 11 | RchiOBHmChr6g0251781 | [Chr6](https://lipm-browsers.toulouse.inra.fr/pub/RchiOBHm-V2/jbrowse/current/?data=../data/RchiOBHm-V2&loc=RchiOBHmChr6:7039197..7049078&highlight=RchiOBHmChr6:7044197..7044078) | 19 | 135 | promoter | Heavy metal-associated domain, HMA | Metal ion binding protein | (Manara et al., 2020) |
| 12 | RchiOBHmChr5g0009251 | [Chr5](https://lipm-browsers.toulouse.inra.fr/pub/RchiOBHm-V2/jbrowse/current/?data=../data/RchiOBHm-V2&loc=RchiOBHmChr5:6171422..6181306&highlight=RchiOBHmChr5:6176422..6176306) | 19 | 135 | Intergenic | Transmembrane protein, | Uncharacterized protein |  |
| 13 | RchiOBHmChr5g0009241 | [Chr5](https://lipm-browsers.toulouse.inra.fr/pub/RchiOBHm-V2/jbrowse/current/?data=../data/RchiOBHm-V2&loc=RchiOBHmChr5:6171422..6181306&highlight=RchiOBHmChr5:6176422..6176306) | 19 | 135 | Intergenic | L-3-cyanoalanine synthase 2 | Uncharacterized protein |  |
| 14 | RchiOBHmChr1g0325641 | [Chr1](https://lipm-browsers.toulouse.inra.fr/pub/RchiOBHm-V2/jbrowse/current/?data=../data/RchiOBHm-V2&loc=RchiOBHmChr1:13609795..13619911&highlight=RchiOBHmChr1:13614795..13614911) | 20 | 135 | promoter | Protein ENHANCED DISEASE RESISTANCE 2, START | Disease resistance protein | (Tang et al., 2005) |
| 15 | RchiOBHmChr7g0219521 | [Chr7](https://lipm-browsers.toulouse.inra.fr/pub/RchiOBHm-V2/jbrowse/current/?data=../data/RchiOBHm-V2&loc=RchiOBHmChr7:38037324..38047214&highlight=RchiOBHmChr7:38042324..38042214) | 28 | 135 | Intergenic | Carboxylesterase | Transferase | (Langenbach et al., 2013) |
| 16 | RchiOBHmChr7g0219511 | [Chr7](https://lipm-browsers.toulouse.inra.fr/pub/RchiOBHm-V2/jbrowse/current/?data=../data/RchiOBHm-V2&loc=RchiOBHmChr7:38037324..38047214&highlight=RchiOBHmChr7:38042324..38042214) | 28 | 135 | Intergenic | Proteasome ATPase | ATPase | (Dal Bosco et al., 2004) |
| 17 | RchiOBHmChr7g0180521 | [Chr7](https://lipm-browsers.toulouse.inra.fr/pub/RchiOBHm-V2/jbrowse/current/?data=../data/RchiOBHm-V2&loc=RchiOBHmChr7:2420280..2430167&highlight=RchiOBHmChr7:2425280..2425167) | 28 | 135 | promoter | RNA-directed DNA polymerase | Protein kinase | (Shiu and Bleecker, 2001) |
| 18 | RchiOBHmChr6g0247521 | [Chr6](https://lipm-browsers.toulouse.inra.fr/pub/RchiOBHm-V2/jbrowse/current/?data=../data/RchiOBHm-V2&loc=RchiOBHmChr6:3446037..3456144&highlight=RchiOBHmChr6:3451037..3451144) | 28 | 135 | Intergenic | Pleiotropic drug resistance protein 1-like | Uncharacterized protein |  |
| 19 | RchiOBHmChr6g0247531 | [Chr6](https://lipm-browsers.toulouse.inra.fr/pub/RchiOBHm-V2/jbrowse/current/?data=../data/RchiOBHm-V2&loc=RchiOBHmChr6:3446037..3456144&highlight=RchiOBHmChr6:3451037..3451144) | 28 | 135 | Intergenic | Abhydrolase domain-containing protein DDB_G0269086-like | Uncharacterized protein |  |
| 20 | RchiOBHmChr2g0139131 | [Chr2](https://lipm-browsers.toulouse.inra.fr/pub/RchiOBHm-V2/jbrowse/current/?data=../data/RchiOBHm-V2&loc=RchiOBHmChr2:56754467..56764362&highlight=RchiOBHmChr2:56759467..56759362) | 28 | 134 | promoter | O-methyltransferase COMT-type, S-adenosyl-L-methionine-dependent methyltransferase | Transferase | (Byeon et al., 2016) |
| 21 | RchiOBHmChr4g0394291 | [Chr4](https://lipm-browsers.toulouse.inra.fr/pub/RchiOBHm-V2/jbrowse/current/?data=../data/RchiOBHm-V2&loc=RchiOBHmChr4:9864084..9874188&highlight=RchiOBHmChr4:9869084..9869188) | 28 | 131 | promoter | Transcription factor WD40-like family | Transcription factor | (Van Leene et al., 2010) |
| 22 | RchiOBHmChr7g0203171 | [Chr7](https://lipm-browsers.toulouse.inra.fr/pub/RchiOBHm-V2/jbrowse/current/?data=../data/RchiOBHm-V2&loc=RchiOBHmChr7:20845058..20854954&highlight=RchiOBHmChr7:20850058..20849954) | 36 | 135 | promoter | Methyltransferase | Transferase | (Niu et al., 2007) |
| 23 | RchiOBHmChr6g0247571 | [Chr6](https://lipm-browsers.toulouse.inra.fr/pub/RchiOBHm-V2/jbrowse/current/?data=../data/RchiOBHm-V2&loc=RchiOBHmChr6:3515077..3525176&highlight=RchiOBHmChr6:3520077..3520176) | 36 | 135 | promoter | Proteasome ATPase | ATPase | (Brenner et al., 2009) |
| 24 | RchiOBHmChr2g0175591 | [Chr2](https://lipm-browsers.toulouse.inra.fr/pub/RchiOBHm-V2/jbrowse/current/?data=../data/RchiOBHm-V2&loc=RchiOBHmChr2:87729570..87739661&highlight=RchiOBHmChr2:87734570..87734661) | 40 | 130 | promoter | Ribonucleoside-diphosphate reductase | Reductase | (Doblas et al., 2013) |
| 25 | RchiOBHmChr5g0020641 | [Chr5](https://lipm-browsers.toulouse.inra.fr/pub/RchiOBHm-V2/jbrowse/current/?data=../data/RchiOBHm-V2&loc=RchiOBHmChr5:14899423..14909510&highlight=RchiOBHmChr5:14904423..14904510) | 50 | 135 | 5’UTR | Phenylalanine--tRNA ligase | tRNA ligase | (Duchêne et al., 2005) |
| 26 | RchiOBHmChr7g0235131 | [Chr7](https://lipm-browsers.toulouse.inra.fr/pub/RchiOBHm-V2/jbrowse/current/?data=../data/RchiOBHm-V2&loc=RchiOBHmChr7:59791181..59801265&highlight=RchiOBHmChr7:59796181..59796265) | 52 | 136 | promoter | Transcription factor TIFY family | Transcription factor | (Vanholme et al., 2007) |
| 27 | RchiOBHmChr3g0470211 | [Chr3](https://lipm-browsers.toulouse.inra.fr/pub/RchiOBHm-V2/jbrowse/current/?data=../data/RchiOBHm-V2&loc=RchiOBHmChr3:30212842..30222720&highlight=RchiOBHmChr3:30217842..30217720) | 56 | 135 | promoter | Protein CLAVATA 3-like | Uncharacterized protein |  |
| 28 | RchiOBHmChr1g0345961 | [Chr1](https://lipm-browsers.toulouse.inra.fr/pub/RchiOBHm-V2/jbrowse/current/?data=../data/RchiOBHm-V2&loc=RchiOBHmChr1:38141348..38151272&highlight=RchiOBHmChr1:38146348..38146272) | 65 | 139 | Intergenic | DNA-directed RNA polymerase | Transcription factor | (Ream et al., 2009) |
| 29 | RchiOBHmChr1g0345971 | [Chr1](https://lipm-browsers.toulouse.inra.fr/pub/RchiOBHm-V2/jbrowse/current/?data=../data/RchiOBHm-V2&loc=RchiOBHmChr1:38141348..38151272&highlight=RchiOBHmChr1:38146348..38146272) | 65 | 139 | Intergenic | PGG domain-containing protein | Uncharacterized protein |  |
| 30 | RchiOBHmChr6g0293581 | [Chr6](https://lipm-browsers.toulouse.inra.fr/pub/RchiOBHm-V2/jbrowse/current/?data=../data/RchiOBHm-V2&loc=RchiOBHmChr6:56210080..56220008&highlight=RchiOBHmChr6:56215080..56215008) | 65 | 135 | 5’UTR | Benzodiazapine receptor (BZRP) | Uncharacterized protein |  |
| 31 | RchiOBHmChr4g0387681 | [Chr4](https://lipm-browsers.toulouse.inra.fr/pub/RchiOBHm-V2/jbrowse/current/?data=../data/RchiOBHm-V2&loc=RchiOBHmChr4:2903611..2913683&highlight=RchiOBHmChr4:2908611..2908683) | 65 | 135 | Intergenic | hypothetical protein | Uncharacterized protein |  |
| 32 | RchiOBHmChr4g0387671 | [Chr4](https://lipm-browsers.toulouse.inra.fr/pub/RchiOBHm-V2/jbrowse/current/?data=../data/RchiOBHm-V2&loc=RchiOBHmChr4:2903611..2913683&highlight=RchiOBHmChr4:2908611..2908683) | 65 | 135 | Intergenic | hypothetical protein | Uncharacterized protein |  |
| 33 | RchiOBHmChr2g0088251 | [Chr2](https://lipm-browsers.toulouse.inra.fr/pub/RchiOBHm-V2/jbrowse/current/?data=../data/RchiOBHm-V2&loc=RchiOBHmChr2:2889347..2899419&highlight=RchiOBHmChr2:2894347..2894419) | 65 | 135 | promoter | F-box domain, leucine-rich repeat domain, L domain-containing protein | EIN3-binding F-box protein | (An et al., 2010) |
| 34 | RchiOBHmChr2g0094101 | [Chr2](https://lipm-browsers.toulouse.inra.fr/pub/RchiOBHm-V2/jbrowse/current/?data=../data/RchiOBHm-V2&loc=RchiOBHmChr2:7140350..7150278&highlight=RchiOBHmChr2:7145350..7145278) | 65 | 135 | 3’UTR | Ribosomal protein L18ae family | Uncharacterized protein |  |
| 35 | RchiOBHmChr2g0094111 | [Chr2](https://lipm-browsers.toulouse.inra.fr/pub/RchiOBHm-V2/jbrowse/current/?data=../data/RchiOBHm-V2&loc=RchiOBHmChr2:7140350..7150278&highlight=RchiOBHmChr2:7145350..7145278) | 65 | 135 | promoter | Zinc finger, C2H2, LYAR-type | Metal ion binding protein | (Manara et al., 2020) |
| 36 | RchiOBHmChr1g0330541 | [Chr1](https://lipm-browsers.toulouse.inra.fr/pub/RchiOBHm-V2/jbrowse/current/?data=../data/RchiOBHm-V2&loc=RchiOBHmChr1:20324678..20334606&highlight=RchiOBHmChr1:20329678..20329606) | 65 | 135 | 3’UTR | FBD domain-containing protein | Uncharacterized protein |  |
| 37 | RchiOBHmChr1g0356211 | [Chr1](https://lipm-browsers.toulouse.inra.fr/pub/RchiOBHm-V2/jbrowse/current/?data=../data/RchiOBHm-V2&loc=RchiOBHmChr1:48745567..48755639&highlight=RchiOBHmChr1:48750567..48750639) | 65 | 135 | promoter | Protein kinase RLK-Pelle-LRR-XII-1 family | Protein kinase | (Shiu and Bleecker, 2001) |
| 38 | RchiOBHmChr3g0479331 | [Chr3](https://lipm-browsers.toulouse.inra.fr/pub/RchiOBHm-V2/jbrowse/current/?data=../data/RchiOBHm-V2&loc=RchiOBHmChr3:30212842..30222720&highlight=RchiOBHmChr3:30217842..30217720) | 65 | 134 | promoter | PMR5 domain, PC-Esterase, trichome birefringence/trichome birefringence-like 1 | Uncharacterized protein |  |
| 39 | RchiOBHmChr3g0452981 | [Chr3](https://lipm-browsers.toulouse.inra.fr/pub/RchiOBHm-V2/jbrowse/current/?data=../data/RchiOBHm-V2&loc=RchiOBHmChr3:3508776..3518840&highlight=RchiOBHmChr3:3513776..3513840) | 68 | 132 | Intergenic | Frigida-like protein | Uncharacterized protein |  |
| 40 | RchiOBHmChr3g0452971 | [Chr3](https://lipm-browsers.toulouse.inra.fr/pub/RchiOBHm-V2/jbrowse/current/?data=../data/RchiOBHm-V2&loc=RchiOBHmChr3:3508776..3518840&highlight=RchiOBHmChr3:3513776..3513840) | 68 | 132 | Intergenic | Frigida-like protein | Uncharacterized protein |  |
| 41 | RchiOBHmChr3g0485021 | [Chr3](https://lipm-browsers.toulouse.inra.fr/pub/RchiOBHm-V2/jbrowse/current/?data=../data/RchiOBHm-V2&loc=RchiOBHmChr3:31595111..31605176&highlight=RchiOBHmChr3:31600111..31600176) | 70 | 133 | promoter | Histone deacetylation protein Rxt3 | Histone deacetylation protein | (Perrella et al., 2016) |
| 42 | RchiOBHmChr3g0464641 | [Chr3](https://lipm-browsers.toulouse.inra.fr/pub/RchiOBHm-V2/jbrowse/current/?data=../data/RchiOBHm-V2&loc=RchiOBHmChr3:25155450..25165381&highlight=RchiOBHmChr3:25160450..25160381) | 78 | 135 | promoter | E3 ubiquitin-protein ligase RLIM-like protein | Uncharacterized protein |  |
| 43 | RchiOBHmChr4g0437391 | [Chr4](https://lipm-browsers.toulouse.inra.fr/pub/RchiOBHm-V2/jbrowse/current/?data=../data/RchiOBHm-V2&loc=RchiOBHmChr4:60489625..60499677&highlight=RchiOBHmChr4:60494625..60494677) | 79 | 130 | intron | Flavonol 3-sulfotransferase | Transferase | (Klein and Papenbrock, 2004) |
| 44 | RchiOBHmChr4g0444591 | [Chr4](https://lipm-browsers.toulouse.inra.fr/pub/RchiOBHm-V2/jbrowse/current/?data=../data/RchiOBHm-V2&loc=RchiOBHmChr4:65251662..65261709&highlight=RchiOBHmChr4:65256662..65256709) | 83 | 130 | 3’UTR | N-hydroxythioamide S-beta-glucosyltransferase | Transferase | (Kopycki et al., 2013) |
| 45 | RchiOBHmChr5g0013371 | [Chr5](https://lipm-browsers.toulouse.inra.fr/pub/RchiOBHm-V2/jbrowse/current/?data=../data/RchiOBHm-V2&loc=RchiOBHmChr5:9036768..9046806&highlight=RchiOBHmChr5:9041768..9041806) | 97 | 135 | promoter | Glucan endo-1,3-beta-D-glucosidase | Glucosidase | (Kim et al., 2006) |
| 46 | RchiOBHmChr5g0013381 | [Chr5](https://lipm-browsers.toulouse.inra.fr/pub/RchiOBHm-V2/jbrowse/current/?data=../data/RchiOBHm-V2&loc=RchiOBHmChr5:9036768..9046806&highlight=RchiOBHmChr5:9041768..9041806) | 97 | 135 | promoter | Glucan endo-1,3-beta-D-glucosidase | Glucosidase | (Kim et al., 2006) |
| 47 | RchiOBHmChr2g0105281 | [Chr2](https://lipm-browsers.toulouse.inra.fr/pub/RchiOBHm-V2/jbrowse/current/?data=../data/RchiOBHm-V2&loc=RchiOBHmChr2:16603874..16613913&highlight=RchiOBHmChr2:16608874..16608913) | 97 | 135 | promoter | Pentatricopeptide | Transcription factor | (Lurin et al., 2004) |

**Reference:**

Abdel-Ghany, S.E., and Pilon, M. (2008). MicroRNA-mediated systemic down-regulation of copper protein expression in response to low copper availability in Arabidopsis. *J Biol Chem* 283(23)**,** 15932-15945. doi: 10.1074/jbc.M801406200.

An, F., Zhao, Q., Ji, Y., Li, W., Jiang, Z., Yu, X., et al. (2010). Ethylene-induced stabilization of ETHYLENE INSENSITIVE3 and EIN3-LIKE1 is mediated by proteasomal degradation of EIN3 binding F-box 1 and 2 that requires EIN2 in Arabidopsis. *Plant Cell* 22(7)**,** 2384-2401. doi: 10.1105/tpc.110.076588.

Ashfield, T., Ong, L.E., Nobuta, K., Schneider, C.M., and Innes, R.W. (2004). Convergent evolution of disease resistance gene specificity in two flowering plant families. *Plant Cell* 16(2)**,** 309-318. doi: 10.1105/tpc.016725.

Brenner, E.D., Feinberg, P., Runko, S., and Coruzzi, G.M. (2009). A mutation in the Proteosomal Regulatory Particle AAA-ATPase-3 in Arabidopsis impairs the light-specific hypocotyl elongation response elicited by a glutamate receptor agonist, BMAA. *Plant Mol Biol* 70(5)**,** 523-533. doi: 10.1007/s11103-009-9489-7.

Bundock, P., and Hooykaas, P. (2005). An Arabidopsis hAT-like transposase is essential for plant development. *Nature* 436(7048)**,** 282-284. doi: 10.1038/nature03667.

Byeon, Y., Lee, H.J., Lee, H.Y., and Back, K. (2016). Cloning and functional characterization of the Arabidopsis N-acetylserotonin O-methyltransferase responsible for melatonin synthesis. *J Pineal Res* 60(1)**,** 65-73. doi: 10.1111/jpi.12289.

Dal Bosco, C., Lezhneva, L., Biehl, A., Leister, D., Strotmann, H., Wanner, G., et al. (2004). Inactivation of the chloroplast ATP synthase gamma subunit results in high non-photochemical fluorescence quenching and altered nuclear gene expression in Arabidopsis thaliana. *J Biol Chem* 279(2)**,** 1060-1069. doi: 10.1074/jbc.M308435200.

Doblas, V.G., Amorim-Silva, V., Posé, D., Rosado, A., Esteban, A., Arró, M., et al. (2013). The SUD1 gene encodes a putative E3 ubiquitin ligase and is a positive regulator of 3-hydroxy-3-methylglutaryl coenzyme a reductase activity in Arabidopsis. *Plant Cell* 25(2)**,** 728-743. doi: 10.1105/tpc.112.108696.

Duchêne, A.M., Giritch, A., Hoffmann, B., Cognat, V., Lancelin, D., Peeters, N.M., et al. (2005). Dual targeting is the rule for organellar aminoacyl-tRNA synthetases in Arabidopsis thaliana. *Proc Natl Acad Sci U S A* 102(45)**,** 16484-16489. doi: 10.1073/pnas.0504682102.

Job, G., Brugger, C., Xu, T., Lowe, B.R., Pfister, Y., Qu, C., et al. (2016). SHREC Silences Heterochromatin via Distinct Remodeling and Deacetylation Modules. *Mol Cell* 62(2)**,** 207-221. doi: 10.1016/j.molcel.2016.03.016.

Kim, J., Shiu, S.H., Thoma, S., Li, W.H., and Patterson, S.E. (2006). Patterns of expansion and expression divergence in the plant polygalacturonase gene family. *Genome Biol* 7(9)**,** R87. doi: 10.1186/gb-2006-7-9-r87.

Klein, M., and Papenbrock, J. (2004). The multi-protein family of Arabidopsis sulphotransferases and their relatives in other plant species. *J Exp Bot* 55(404)**,** 1809-1820. doi: 10.1093/jxb/erh183.

Kopycki, J., Wieduwild, E., Kohlschmidt, J., Brandt, W., Stepanova, A.N., Alonso, J.M., et al. (2013). Kinetic analysis of Arabidopsis glucosyltransferase UGT74B1 illustrates a general mechanism by which enzymes can escape product inhibition. *Biochem J* 450(1)**,** 37-46. doi: 10.1042/bj20121403.

Langenbach, C., Campe, R., Schaffrath, U., Goellner, K., and Conrath, U. (2013). UDP-glucosyltransferase UGT84A2/BRT1 is required for Arabidopsis nonhost resistance to the Asian soybean rust pathogen Phakopsora pachyrhizi. *New Phytol* 198(2)**,** 536-545. doi: 10.1111/nph.12155.

Lurin, C., Andrés, C., Aubourg, S., Bellaoui, M., Bitton, F., Bruyère, C., et al. (2004). Genome-wide analysis of Arabidopsis pentatricopeptide repeat proteins reveals their essential role in organelle biogenesis. *Plant Cell* 16(8)**,** 2089-2103. doi: 10.1105/tpc.104.022236.

Manara, A., Fasani, E., Molesini, B., and DalCorso, G. (2020). The Tomato Metallocarboxypeptidase Inhibitor I, which Interacts with a Heavy Metal-Associated Isoprenylated Protein, Is Implicated in Plant Response to Cadmium. 25(3). doi: 10.3390/molecules25030700.

Niu, L., Lu, F., Pei, Y., Liu, C., and Cao, X. (2007). Regulation of flowering time by the protein arginine methyltransferase AtPRMT10. *EMBO Rep* 8(12)**,** 1190-1195. doi: 10.1038/sj.embor.7401111.

Perrella, G., Carr, C., Asensi-Fabado, M.A., Donald, N.A., and Páldi, K. (2016). The Histone Deacetylase Complex 1 Protein of Arabidopsis Has the Capacity to Interact with Multiple Proteins Including Histone 3-Binding Proteins and Histone 1 Variants. 171(1)**,** 62-70. doi: 10.1104/pp.15.01760.

Plong, A., Rodriguez, K., Alber, M., and Chen, W. (2021). CLAVATA3 mediated simultaneous control of transcriptional and post-translational processes provides robustness to the WUSCHEL gradient. 12(1)**,** 6361. doi: 10.1038/s41467-021-26586-0.

Ream, T.S., Haag, J.R., Wierzbicki, A.T., Nicora, C.D., Norbeck, A.D., Zhu, J.K., et al. (2009). Subunit compositions of the RNA-silencing enzymes Pol IV and Pol V reveal their origins as specialized forms of RNA polymerase II. *Mol Cell* 33(2)**,** 192-203. doi: 10.1016/j.molcel.2008.12.015.

Shiu, S.H., and Bleecker, A.B. (2001). Receptor-like kinases from Arabidopsis form a monophyletic gene family related to animal receptor kinases. *Proc Natl Acad Sci U S A* 98(19)**,** 10763-10768. doi: 10.1073/pnas.181141598.

Tang, D., Ade, J., Frye, C.A., and Innes, R.W. (2005). Regulation of plant defense responses in Arabidopsis by EDR2, a PH and START domain-containing protein. *Plant J* 44(2)**,** 245-257. doi: 10.1111/j.1365-313X.2005.02523.x.

Van Leene, J., Hollunder, J., Eeckhout, D., Persiau, G., Van De Slijke, E., Stals, H., et al. (2010). Targeted interactomics reveals a complex core cell cycle machinery in Arabidopsis thaliana. *Mol Syst Biol* 6**,** 397. doi: 10.1038/msb.2010.53.

Vanholme, B., Grunewald, W., Bateman, A., Kohchi, T., and Gheysen, G. (2007). The tify family previously known as ZIM. *Trends Plant Sci* 12(6)**,** 239-244. doi: 10.1016/j.tplants.2007.04.004.

Wang, Y., Zhang, W.Z., Song, L.F., Zou, J.J., Su, Z., and Wu, W.H. (2008). Transcriptome analyses show changes in gene expression to accompany pollen germination and tube growth in Arabidopsis. *Plant Physiol* 148(3)**,** 1201-1211. doi: 10.1104/pp.108.126375.

**Supplementary Table S2** Different rose varieties were used in this study

| No. | Variety name | Scientific name | Bred country | Petal color |
| --- | --- | --- | --- | --- |
| 1 | Slater’s crimson China | *Rosa chinensis* | China | red |
| 2 | Blue River | *R.chinensis* | Germany | red |
| 3 | Betty Prior | *R.hybrida* | Unkown | red |
| 4 | Dortmund | *R.hybrida* | Germany | red |
| 5 | Uncle Walter | *R.hybrida* | UK | red |
| 6 | Pierre de Ronasard | *R.hybrida* | France | red |
| 7 | Hiohgi | *R.hybrida* | Japan | red |
| 8 | Hohoemi | *R.hybrid* | Japan | red |
| 9 | Red Success | *R.hybrid* | France | red |
| 10 | Terrazza Voila | *R.hybrid* | Netherlands | red |
| 11 | Wonderful Wife | *R.hybrid* | UK | red |
| 12 | Crimson Glory | *R.hybrid* | Germany | red |
| 13 | Red Cap | *R.hybrid* | United State | red |
| 14 | Carola | *R.hybrida* | Germany | red |
| 15 | Seba | *R.hybrida* | Unkown | red |
| 16 | Black Lady | *R.hybrida* | Germany | red |
| 17 | Gold Carriage | *R.hybrida* | China | red |
| 18 | Zajibiaoyan | *R.hybrida* | China | red |
| 19 | Hana-Busa | *R.hybrida* | Japan | red |
| 20 | Huangjiabaxinuo | *R.hybrida* | China | red |
| 21 | 2018-08-3 | *R.hybrida* | China | red |
| 22 | Burgundy Iceberg | *R.hybrida* | Australia | red |
| 23 | Xiangchun | *R.hybrida* | China | red |
| 24 | Dongfanghong | *R.hybrida* | China | red |
| 25 | Cherry bonica | *R.hybrida* | France | red |
| 26 | Red Lace | *R.hybrida* | Netherlands | red |
| 27 | Rhapsody in Blue | *R.hybrida* | UK | red |
| 28 | Ingrid Bergman | *R.hybrida* | Denmark | red |
| 29 | Viridiflora | *R.chinensis* | China | green |
| 30 | Green Star | *R.hybrida* | China | green |
| 31 | Lvye | *R.hybrida* | China | green |
| 32 | Eclair | *R.hybrida* | Japan | green |
| 33 | Creamy Eden | *R.hybrida* | France | green |
| 34 | Golden Celebration | *R.hybrida* | UK | yellow |
| 35 | Yellow Meilove | *R.hybrida* | France | yellow |
| 36 | Adolf Horstmann | *R.hybrid* | Germany | yellow |
| 37 | Golden scepter | *R.hybrida* | Netherlands | yellow |
| 38 | Kent Princess | *R.hybrida* | UK | yellow |
| 39 | Oregold | *R.hybrida* | Germany | yellow |
| 40 | Gold Bunny | *R.hybrida* | France | yellow |
| 41 | Australian Gold | *R.hybrida* | Germany | yellow |
| 42 | Golden Shower | *R.hybrida* | United State | yellow |
| 43 | Baihe | *R.hybrida* | China | white |
| 44 | 2018-31-117 | *R.hybrida* | China | white |
| 45 | White Ohara | *R.hybrida* | Japan | white |
| 46 | Bridal White | *R.hybrida* | United State | white |
| 47 | Lvyun | *R.hybrida* | China | white |
| 48 | Beizhi | *R.hybrida* | China | white |
| 49 | Snowflake | *R.hybrida* | Netherlands | white |
| 50 | White Satin | *R.hybrida* | United State | white |
| 51 | Tiantanbai | *R.hybrida* | China | white |

**Supplementary Table S3** The sequences list of primers used in this study

| Gene name | Purpose | Primer Sequence (5'→3') | TM  (℃) | Accession number |
| --- | --- | --- | --- | --- |
| *RcMYB114* | gene clone | F: CAACGTCATTAACTGTGGGATC | 55 | MW239568 |
|  |  | R: GCCGTGAGCAGTGGCTTTC |  |  |
| *pRcMYB114^Red^* | promoter clone and GUS assay | F:ACGCgtcgacGGAAACTGAAGAATTGGAATCATCGGAC | 58 | RchiOBHmChr7g0235271 |
|  |  | R:ACGCgtcgacTTTTGTTGACTCACGTACTTATTCTGAAC |  |  |
| *RcMYB114^Red^* | transient expression | F:GAAATTtctagaATGGAGGACCAGTCGGGTTTGAG | 58 | MW239568 |
|  |  | R: GAAATTgagctcTCATTATCGATCTAAGAATGTCATCC |  |  |
| *RcbHLH* |  | F:GAAATTtcgcgaATGGCTACACCGCCACCGAGTAGTAGC | 57 | KY783912 |
|  |  | R: GAAATTaccggtTTAAGAGTCAGATTGGGGTATCAC |  |  |
| *RcMYB114^Red^* | Subcellular localization of *RcMYB114* | F:GAAATTctcgagATGGAGGACCAGTCGGGTTTGAG | 58 | MW239569 |
|  |  | R: GAAATTgaattcTCGATCTAAGAATGTCATCCA |  |  |
| *Rosa1* | Transposable-Element-like marker | F: CGGAAGTCCTTGTTCTGACCTGTTGGTCTGACG | 58 | MW430097 |
|  |  | R: GCTAACCAGCCCTATTCGCGCCCCTATATG |  |  |
| *RcMYB114^Red^ a:AD* | Yeast two-hybrid assays | F: GAAATTcatatgATGGAGGACCAGTCGGGTTTGAG | 58 | MW239569 |
|  |  | R: GAAATTgaattcTCATTATCGATCTAAGAATGTCATCC |  |  |
| *RcMYB114^Red^:BK* |  | F: GAAATTcatatgATGGAGGACCAGTCGGGTTTGAG | **58** | MW239569 |
|  |  | R: GAAATTgaattcTCATTATCGATCTAAGAATGTCATCC |  |  |
| *RcWD40:AD* and *RcWD40:BK* |  | F: GAAATTcatatgATGGAGAACTCGACCCAAGAATC | 57 | MW239571 |
|  |  | R: GAAATTgaattcTCAAACCTTCAACAGCTGCATCTTA |  |  |
| *RcbHLH:AD* and *RcbHLH:BK* |  | F: GAAATTggatccATGGCTACACCGCCACCGAGTAGTAGC | 57 | KY783912 |
|  |  | R: GAAATTgaattcTCATTAAGAGTCAGATTGGGGTATCAC |  |  |
| *RcMYB114* | RT-qPCR | F:ACCAAGCGGCGTCGGGACAAAC | 60 | MW239569 |
|  |  | R: CCCGTCAAACAGAGTGAACTGGTCG |  |  |
| *RcC4H* |  | F:ATGTTCGACAGGAGATTTGAAAGCG | 60 | RchiOBHmChr5g0073351 |
|  |  | R:ATTATACTCGAAGCTCTGCGCCAAC |  |  |
| *RcCHI* |  | F:TTTCCTCCCGCCGTCAAGCC | 60 | RchiOBHmChr1g0365111 |
|  |  | R:CCAAGTAGACTCCAATCGCCGTGAA |  |  |
| *RcCHS* |  | F:CTACTTTCGTATCACCAACAGCG | 60 | RchiOBHmChr1g0316451 |
|  |  | R:TTCAGTCAAATACATATAACGCTTC |  |  |
| *RcF3H* |  | F:GCTCCAGGACCAAGTCGGTGGACT | 60 | RchiOBHmChr2g0099421 |
|  |  | R:TGATCGGCGTTCTTGAACCTCCC |  |  |
| *RcANS* |  | F:GGAACTTGCCCTCGGCGTGG | 60 | RchiOBHmChr7g0199941 |
|  |  | R:ATGACGATGGAGTTGGGCACGC |  |  |
| *RcPAL* |  | F:AAGATTTTGCGAGAAGGATTTGC | 60 | RchiOBHmChr3g0469861 |
|  |  | R:GCATTCTTCTCATTCTCACCATTTGT |  |  |
| *RcUFGT* |  | F:GCCCCAAACACCCTCTTCTCA | 60 | RchiOBHmChr1g0383951 |
|  |  | R:CCTGAGGCTTACCCACAAAAACA |  |  |
| *RcLAR* |  | F:GCCCTTCTCGTCCTCCCAA | 60 | RchiOBHmChr4g0435881 |
|  |  | R:GCACCACCCACGGCTGAT |  |  |
| *RcFLS* |  | F:GTGAACCACGACATTTCTAACGAG | 60 | RchiOBHmChr6g0295121 |
|  |  | R:CCCTCCACGGACTTAGAGTTCGG |  |  |
| *RcDFR* |  | F:CACCGTGCGAGACCCTGCTAA | 60 | RchiOBHmChr6g0301421 |
|  |  | R:TCAAAATCCATAGGAGTGGCGACA |  |  |
| *RcWD40* |  | F:ACTCGACCCAAGAATCCCACCTC | 60 | MW239571 |
|  |  | R:GTTGGAGAACTCCTCGATGAAGC |  |  |
| *RcAN1* |  | F:AGCCTAAACTGATAGAGGAACCGAC | 60 | RchiOBHmChr6g0291161 |
|  |  | R:TAACAACCCTTCTCTGTACGGGC |  |  |
| *RcAN2* |  | F:ATGGGTGATTTGGGAATGGTAGA | 60 | RchiOBHmChr3g0448721 |
|  |  | R:AGTAACCCAAAGGTCCATGTCCA |  |  |
| *RcActin* |  | F: GGCTGTTCTTTCCCTCTATGC | 58 | KC514920 |
|  |  | R: GCGTTTCAGATGCCCAGAA |  |  |
| *NtCHS* |  | F:ACTCCGGATGGCTAAGGACT | 60 | AF311783 |
|  |  | R:ACCTATAATGACCGCGGCTG |  |  |
| *NtCHI* |  | F:ACTGGCACTGGAAATGCTGT | 60 | AB213651 |
|  |  | R:AAACTGACGCGTCGGCATAG |  |  |
| *NtF3H* |  | F:AAGGCAGTAATGGACGAGC | 60 | AB289450 |
|  |  | R:TGGCCTTCTCAGCAGCTTTT |  |  |
| *NtFLS* |  | F:GGTTAGGAGCCCATGAAATGA | 60 | AB289451 |
|  |  | R:CACAACACCAAGTGCCAAATC |  |  |
| *NtLAR* |  | F:TCAATGGTGCGAAAGGACTC | 60 | AM827419 |
|  |  | R:TGCTGCAGAGAATATCAACC |  |  |
| *NtDFR* |  | F:GGGAATGAAGCTCACTACAGC | 60 | EF421429 |
|  |  | R:ACTCCGGCCATTTCTCTTGG |  |  |
| *NtANS* |  | F:TGTCCCCAACCAGAACTAGC | 60 | AB289447 |
|  |  | R:TTTGCCGTTACCCACTGTCC |  |  |
| *NtUFGT* |  | F:TTTCGGGGACCAAAAGCTGA | 60 | FG627024 |
|  |  | R:CTGAAAAAGGCATCCAATGCAC |  |  |
| *NtGAPDH1* |  | F:CTGCTCACTTGAAGGGTGGT | 60 | AJ42133422 |
|  |  | R:GGGAGCAAGGCAATTTGTGG |  |  |
| *RcC4H* | Chromatin immunoprecipitation assay | F: TGGTGGTCAGCGAAGCATCACAGC | 58 | RchiOBHmChr5g0073351 |
|  |  | R: TAGGATGTGGAGCCCAGTAGGAG |  |  |
| *RcCHI* |  | F: TCGAATTATTCGATATGTGGC | 58 | RchiOBHmChr1g0365111 |
|  |  | R: GTATCCTCGGCTCCTCTTGAAGC |  |  |
| *RcCHS* |  | F: CGTTCTCTCGTCTTACCTAC | 58 | RchiOBHmChr1g0316451 |
|  |  | R:TTCAGTCAAATACATATAACGCTTC |  |  |
| *RcF3H* |  | F: AATAGACTCCGTAGAATCGTC | 58 | RchiOBHmChr2g0099421 |
|  |  | R: ATGCTCCTTATGGCGATGTAC |  |  |
| *RcANS* |  | F: GTCAAACACTGCTTGTATGAGAAGTC | 58 | RchiOBHmChr7g0199941 |
|  |  | R: GTAGTTGGCTGCATGACAATC |  |  |
| *RcPAL* |  | F: GACAAGGTCTCCGATGACTTC | 58 | RchiOBHmChr3g0469861 |
|  |  | R: CTACTCATAAGAGAGCAATGTC |  |  |
| *RcUFGT* |  | F: GAAGGCTACCTTAGTAAGTTGAC | 58 | RchiOBHmChr1g0383951 |
|  |  | R: ACAAATGCTGGACTCGTAGCGTAG |  |  |
| *RcLAR* |  | F: GACTATATAATCAGTATTCAC | 58 | RchiOBHmChr4g0435881 |
|  |  | R: CAGAGACATGATCAGCTAACCAG |  |  |
| *RcFLS* |  | F: TGTCCAACAGCAACAGTTGATGC | 58 | RchiOBHmChr6g0295121 |
|  |  | R: GGTGCTAATCATGAGTGATGATC |  |  |
| *RcDFR* |  | F: TCGGGCTTCTAGTCCCAGTGC | 58 | RchiOBHmChr6g0301421 |
|  |  | R: GAGGAGAACTTTGGCAACAGAC |  |  |
| *H3K9* |  | F: CTCGATGTCGTATTCGCTGA | 58 | AT4G03770 |
|  |  | R: GCAACCTATCAACGCTTCGT |  |  |

F, forward primer; R, reverse primer. Underlined characters indicate the restriction enzyme cutting site.TM: actual annealing temperature during PCR and RT-qPCR.
